# Identification of circadian clock genes as regulators of immune infiltration in Hepatocellular Carcinoma

**DOI:** 10.7150/jca.71925

**Published:** 2022-09-01

**Authors:** Zhen Zhang, Zicheng Liang, Wenhui Gao, Shuxian Yu, Zongwei Hou, Kexin Li, Puhua Zeng

**Affiliations:** 1 Department of Oncology, Affiliated Hospital of Hunan Academy of Traditional Chinese Medicine, Changsha 410006, P.R. China.; 2 Department of Internal Medicine, College of Integrated Chinese and Western Medicine of Hunan University of Chinese Medicine, Changsha, Hunan 410208, P.R. China.; 3 School of Chinese Medicine, Hunan University of Chinese Medicine, Changsha 410208, P.R. China.

**Keywords:** Hepatocellular Carcinoma, Circadian clock, Immune infiltration, Chrono-immunotherapy

## Abstract

**Background:** Multiple studies have reported that the immune system is under the control of a circadian clock, especially in cancers, but how circadian clock genes shape tumor immune cell infiltration in hepatocellular carcinoma (HCC) remains unclear.

**Methods:** The rhythmicity of circadian clock genes was investigated using the GETx database. The expression and methylation level of circadian clock genes in HCC and paracancerous was evaluated using the GETx and TCGA databases. The differential expression of circadian clock genes in HCC was analyzed using the “limma” package of the R 4.0.4 software. The prognosis of each circadian clock gene was accessed by Kaplan-Meier survival analysis and Cox proportional hazards regression analysis. Quantitative real-time PCR and immunohistochemistry (IHC) was carried out to confirm the results. The relationship between circadian rhythm and immune infiltration in HCC was evaluated using the TIMER database and the CIBERSORT algorithm.

**Results:** In addition to RORA, RORB, and ARNTL2, there was a rhythmic expression of other circadian clock genes in liver tissue. The correlation between the expression of circadian clock genes differed when comparing HCC and liver tissue. HCC patients who express low levels of PER-1and CRY2 had a poor overall survival (OS). In contrast, patients with higher expression of NPAS2 had a poor prognosis. In HCC, the expression of the PER-1, CRY2, and NPAS2 genes was closely related to immune infiltration.

**Conclusion:** Our study indicated the disruption of the expression of circadian clock-regulated genes in HCC and identified PER-1, CRY2, and NPAS2 as independent predictors of survival. These genes may be applied as candidate molecular targets for diagnosis and therapy of HCC.

## Introduction

Hepatocellular carcinoma (HCC) has long been a clinical concern, especially in China, where it is associated with an estimated 1% mortality rate. The incidence rate of HCC is sixth, and the mortality rate is fourth 1. The prevalence was closely related to its late-stage presentation and aggressive nature which caused a median overall survival of <6 months 2. Currently, surgery is the only treatment of HCC. Other treatment methods such as radiotherapy and chemotherapy are not satisfactory 3. Currently, targeted drugs, including sorafenib and regorafenib, were able to improve the prognosis of patients with HCC. However, these drugs still exist a various of problems, including drug resistance, serious side effects and other problems. Therefore, it's imperative to identify new prognostic biomarkers for the treatment of HCC.

Cancer immunotherapy is a strategy to treat solid tumor with mild side effects and improved survival rates. Multiple lines of evidence suggest the tumor microenvironment (TME), mainly made up tumor cells, immune cells, and matrix components, has a crucial effect on tumor growth, invasion, and patient outcomes 4-6. The presence of immune infiltration cells may affect the progression of many tumors and the therapeutic response 7. For example, infiltration of FoxP3+ regulatory T cells (Treg) in TME is a poor prognostic biomarker in HCC, but the densities of CD20+ B cells or CD57+ natural killer (NK) cells in HCC are positively related to the prognosis of HCC 8. Furthermore, the correlation between genes that contribute to the prognosis of HCC and immune infiltration was investigated using transcriptomic data 9, 10.

In mammals, the circadian rhythm is coordinated by an endogenous timing system, which can be synchronized with the 24-hour environmental cycle generated by the earth's rotation. Accumulating evidence shows that about 10% of the human genome is influenced by the circadian clock genes (CCGs) 11-13. Accumulating evidence supports the relationship between incidence of malignancy and chronic circadian clock 14-16. For example, studies have revealed that night shift work, including long-term shift work increase breast or prostate cancer risk 17, 18. In addition, mice with genetic changes in some clock genes were more susceptible to develop cancers, including hepatocellular carcinoma, lung cancer, and ovarian cancer 19, 20. However, to date, the effects of CCGs on HCC and their underlying mechanisms remain unclear.

It has been well demonstrated that the immune system is controlled by the circadian clock, especially in cancers 14. A previous study reported that the CCGs can modulate the TME in kidney renal clear cell carcinoma 21. Another independent group also revealed that CD4+ and CD8+ infiltration is closely influenced by the expression of several CCGs in thoracic cancers 22. To date, it is unclear how CCGs shape tumor immune cell infiltration in HCC.

The recent development of bioinformatics tools has enabled the exploration of the circadian clock throughout the transcriptome in cancer 23. Herein, we investigate the rhythmicity of CCGs in normal liver tissues and used multiple bioinformatics to comprehensively evaluate the expression of mRNA and prognostic values of CCGs in HCC. Further, we explored the potential correlation between circadian clock and immune cell infiltration in HCC.

## Materials and methods

### Study samples and datasets

Information extracted from the Genotype-Tissue Expression database relative to normal liver tissue was downloaded (https://gtexportal.org) 24 to identify the rhythmic nature of CCGs. High-throughput sequencing of fragments per kilobase of transcript per million mapped reads (FPKM) of HCC tissues, including 50 adjacent tissues and 369 tumor tissues, were obtained from a free and publicly available reference for cancer research, The Cancer Genome Atlas (TCGA) (https://cancergenome.nih.gov) 25. The corresponding clinical information for HCC was downloaded from the UCSC Xena database (https://xena.ucsc.edu/) 26. Samples with incomplete information were deleted prior to the analysis. Ultimately, 350 tumor samples and 50 adjacent tissues were selected.

### Methylation level, gene correlation, and differential gene expression analysis

The RNAseq in transcripts per million reads (TPM) format was converted to log_2_. In addition, a comparison of the gene methylation status between liver and tumor tissue was performed in association with a comparison with the corresponding clinical information. A false discovery rate (FDR) < 0.05 was defined as importance differential methylation. Based on HCC RNA-Seq FPKM data, the mRNA expression level of CCGs in HCC compared to that of adjacent was analyzed using the 'limma' package (http:///www.r-project.org/) of R 4.0.4 software. We applied the Wilcox test to analyze differential expression in 50 precancerous samples and 371 tumor tissues. The threshold for parameters was set as: *P* < 0.05.

### Prognostic value analysis of CCGs in HCC

According to the median level of mRNA expression, the patients were separated into two groups, which were named as high-expression group and low-expression group. Differences regarding the influence of two groups of CCGs on OS were explored using Kaplan-Meier survival analysis and the log-rank test. In addition, univariate and multivariate Cox regression analyses were performed to identify whether CCGs could be independent factors for the prognosis of HCC patients, integrating factors from clinical variables. The results are exhibited with the risk ratio (HR) and the 95% confidence interval. Statistical significance was established at a P-value < 0.05. Genes that significantly influenced survival were selected for further analyses.

### Patients and tissue specimens

In this study, we collected 20 HCC tissue and precancerous tissue samples from the Affiliated Hospital of Hunan Academy of Traditional Chinese Medicine between March 2019 and March 2020. The samples were used in accordance with the relevant regulation of the ethics committee of the Affiliated Hospital of the Hunan Academy of Traditional Chinese Medicine. We had carefully screened about the patients. All of the included patients had no serious medical and surgical diseases, including diabetes, hypertension, and other cancers. All of the patients were aged between 18 and 70 years and had adequate function of major organs, including heart, liver and kidneys. All tissue samples were obtained from patients without receiving any medical treatment before surgery. Patients were excluded if they had chemotherapy, radiotherapy or transarterial chemoembolization therapy.

### qRT-PCR and immunohistochemistry staining

Based on the manufacturer's protocol, total tissue RNA was extracted using TRIzol regent (Tiangene, China). Subsequently, the RNA was transcribed into cDNA. Then, with the cDNA as the template, qRT-PCR was carried out. Relative mRNA expression was calculated using the 2^-ΔΔCt^ method. The primers used were as follows ('F', indicates forward; 'R', indicates reverse): GAPDH-F: 5'-ATCCCATCACCATCTTCC-3'; GAPDH-R: 5'-ATGACCCTTTTGGCTCCC; RORC-F: 5'-CTTTTCCGAGGATGAGAT-3'; RORC-R: 5'-ATGCTTTGGCGATGAGAT-3'; NPAS2-F: 5'-TAAAATCCTTTCTTCCCCATA-3'; NPAS2-R: GGCAATAAAACTCTAAATCG; PER1-F: 5'-CAGCCATTCCGCCTAACCC-3'; PER1-R: 5'-TGCCGCGTAGTGAAAATCCTC.

After fixing with 4% PFA, dehydration with graded alcohol solutions, the samples were made transparent with xylene, before being embedded in paraffin. Paraffin tissue sections with a thickness of 4 μm was stained using the DAB Refine kit according to standard immunohistochemistry protocols. A Zeiss microscope was employed for imaging.

### Immune infiltration analysis of CCGs in HCC

The correlation between CCGs and tumor infiltration immune cells (TIIC) was explored by Tumor Immune Estimation Resource (TIMER 2.0 platform http://timer.cistrome.org/) 27, which facilitates access to tumor immunological, clinical, and genomic characteristics. In this study, we used the 'Gene' module to investigate the relationship between the expression of CCGs and the abundances of five degree of immune infiltration. Gene Expression Profiling Interactive Analysis (GEPIA 28, http://gepia.cancer-pku.cn/) is a bioinformatics platform developed for the analysis and processing of transcriptome data from TCGA and GTEx databases. We employed GEPIA to analyze the correlation between CCGs and biomarkers of TIICs 29.

### Statistical analysis

The results were evaluated using R software 4.0.3 (https://www.r-project.org/). The HTSeq FPKM mRNA data were analyzed using Perl 5.3.0 software (https://www.perl.org/). The Student's t-test was used to identify the methylation difference between tumor and normal tissue. The Pearson correlation coefficient was employed to explore the correlation between circadian clock genes.

## Results

### Definition of core CCGs and expression of these genes in normal liver tissue and HCC

**Figure 1:**
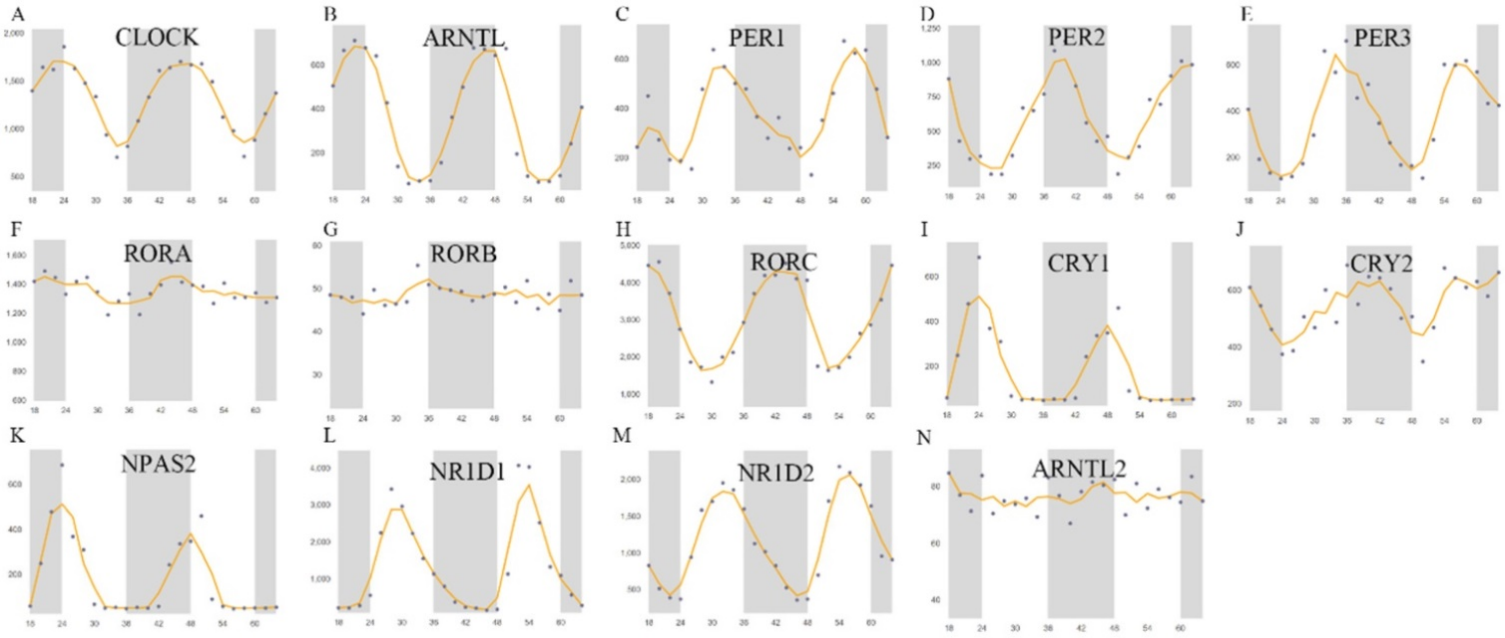
Core CCGs in mouse liver tissue. The circadian rhythm of core circadian clock genes. A. CLOCK; B. ARNTL C. PER1; D. PER2; E. PER3; F. RORA; G. RORB; H. RORC; I. CRYA; J. CRY2; K. NPAS2; L. NR1D1; M. NR1D2; N. ARNTL2.

In this study, 14 previously described genes were selected as the core circadian clock genes, namely CLOCK, NPAS2, ARNTL, ARNTL2, CRY1, CRY2, PER-1, PER-2, PER-3, NR1D1, NR1D2, RORA, RORB, RORC 30, 31. In liver tissues, the expression profile of these genes at different intervals was used to investigate changes in the fluctuation of the expression of these genes. As shown in Figure 1, the rhythmic expression of RORA, RORB, and ARNTL2 was not obvious, while the expression of other genes was more marked. The rhythmic expression of CLOCK, NPAS2, ARNTL, and CRY1 was synchronous, the peak of which appeared at about 00:00 and the trough of which appeared at about 8:00 am. In contrast, the expression of PER-1, PER-2, PER-3, CRY2, NR1D1, and NR1D2 peaked at about 8:00 and reached the lowest value at about 00:00 midnight. Then, we explored the co-expression of CCGs and methylation levels of these genes in tumor and adjacent tissues.

**Figure 2:**
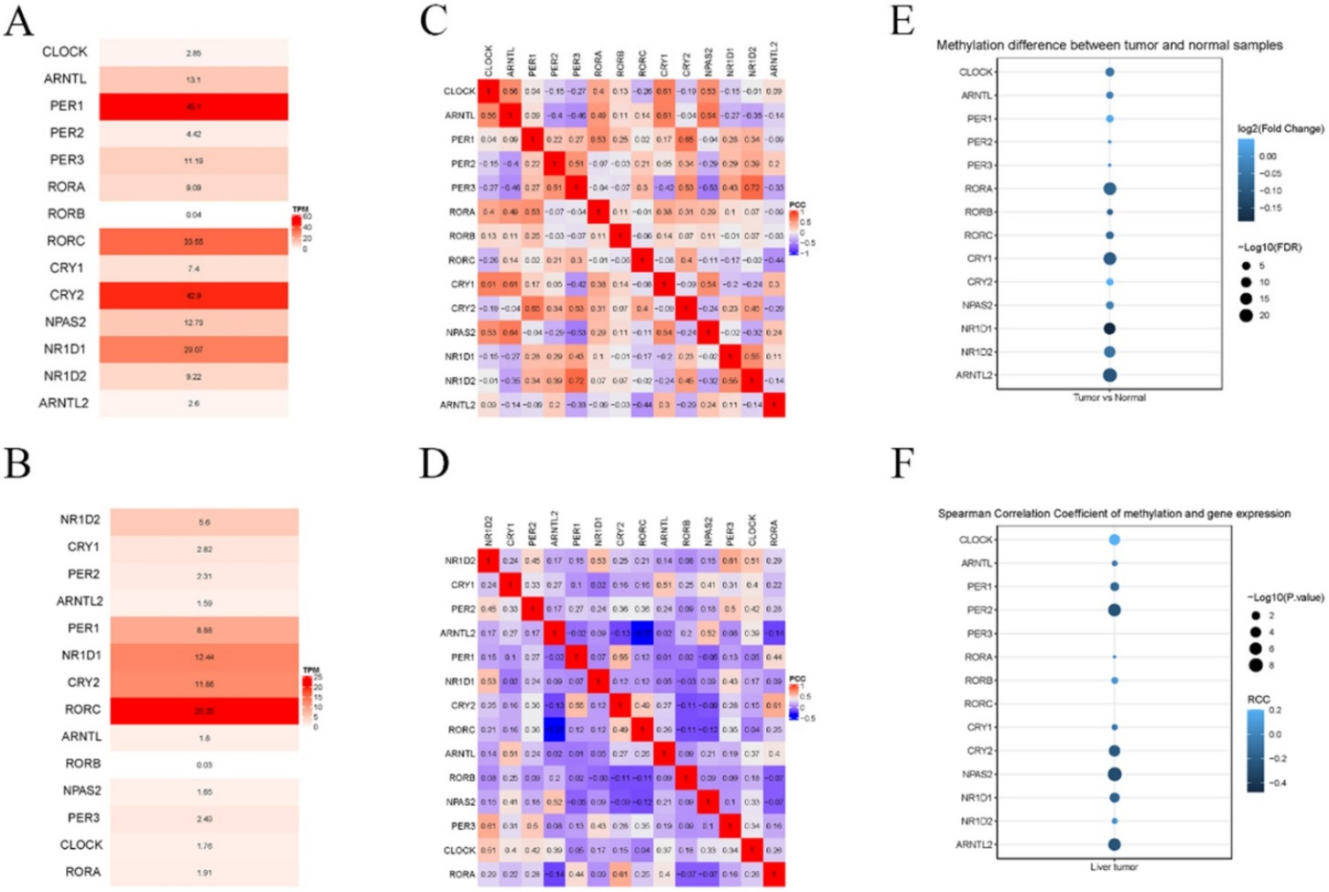
The expression of core CCGs in liver and HCC tissue. (A) Relative expression of CCGs in liver tissues. (B) relative expression of CCGs in tumor tissues. (C) Co-expression of CCGs in normal tissues. (D) Co-expression of CCGs in liver tissues. (E) The methylation difference between tumors and normal tissues. (F) The methylation level affects the core circadian gene expressions.

**Figure 3:**
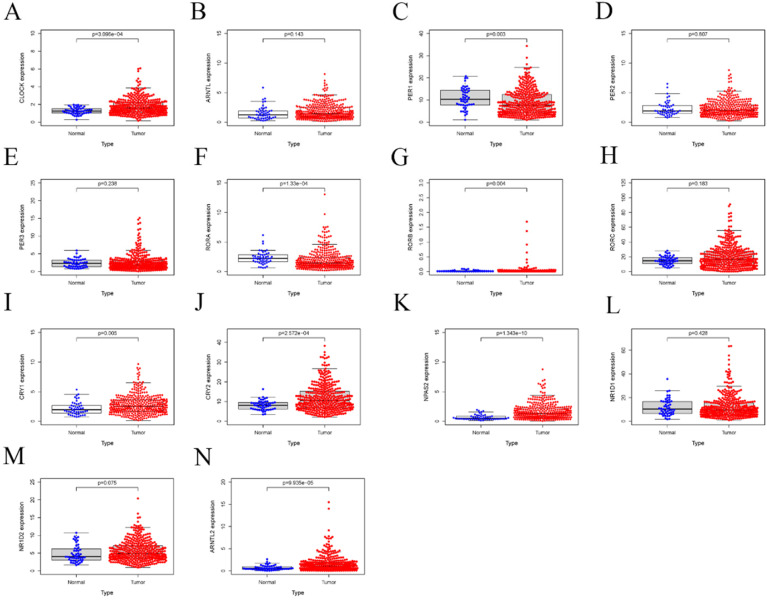
Relative mRNA expression of core CCGs adjacent in and tumor tissues based on TCGA database. A. CLOCK; B. ARNTL C. PER1; D. PER2; E. PER3; F. RORA; G. RORB; H. RORC; I. CRYA; J. CRY2; K. NPAS2; L. NR1D1; M. NR1D2; N. ARNTL2.

Next, we investigated the differential mRNA expression of these genes by comparing HCC and adjacent tissues. As shown in Figure 2, significantly lower expression of RORA, RORC, PER-1, and CRY2 was observed in HCC tissue compared with adjacent tissues, while there was an upregulation of NPAS2, CLOCK, CRY1, CRY2, RORB. Then, the relative expression of these genes was calculated for HCC and adjacent tissues and the result indicated that PER-1 and RORB had the highest and lowest expression in liver tissues, respectively. Co-expression analysis indicated that the correlation between circadian clock gene expression was altered between HCC and liver tissue. Furthermore, we computed the methylation status of genes in HCC and adjacent tissues. As shown in Figure 3, the larger the dot, the darker the color, the more marked the level of gene methylation. The results indicated that over half of CCGs were methylated in HCC, indicating that over half of circadian clock gene expression was downregulated. Altogether these findings indicate that the circadian clock was disrupted in HCC.

Collectively, the results indicated an epigenetic alteration of several CCGs in HCC, including CLOCK, PER-1, RORA, RORB, CRY1, CRY2, NPAS2, NR1D1, and ARNTL2 in HCC, compared to adjacent, which may be potential biomarkers for patients with HCC.

### The prognostic value of differential expression CCGs in HCC

**Figure 4:**
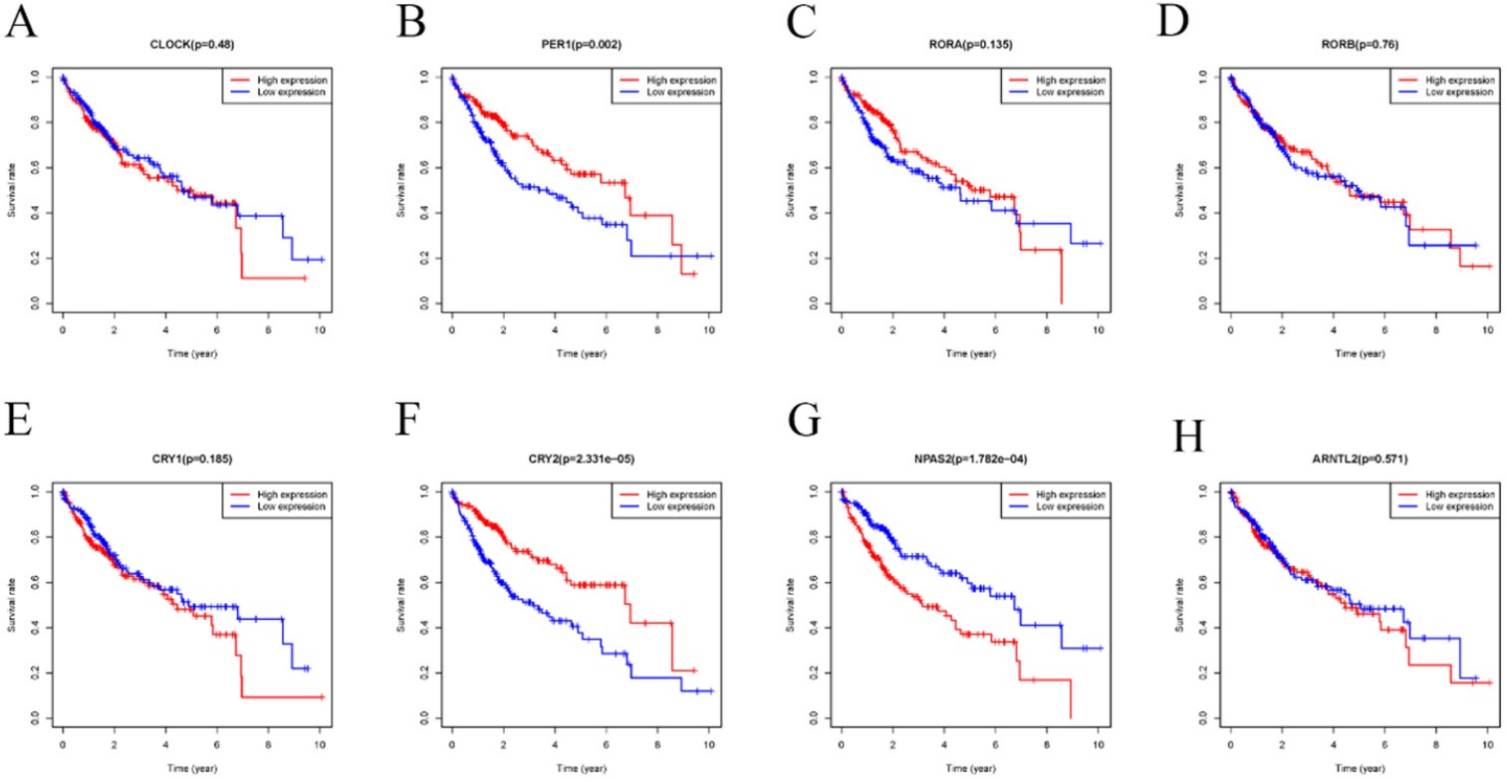
Kaplan-Meier survival analysis of the relation between the differently expressed CCGs in adjacent and tumor tissues and overall survival. A. CLOCK; B. PER1 C. RORA; D. RORB; E. CRY1; F. CRY2; G. NPAS2; H. ARNTL2;

Accumulating evidence has shown that disorder of CCGs were closely associated with tumorigenesis, development, and prognosis of various solid tumors 32, 33. Therefore, we performed a comprehensive evaluation of whether differential expression of CCGs influenced the prognosis of HCC. As shown in Figure 4, HCC patients with low mRNA expression of PER-1 (P=0.002) and CRY2 (P=2.331E-05) had a poor overall survival (OS). In contrast, patients with high expression of NPAS2 (p=1.782E-04) had a poor prognosis.

**Figure 5:**
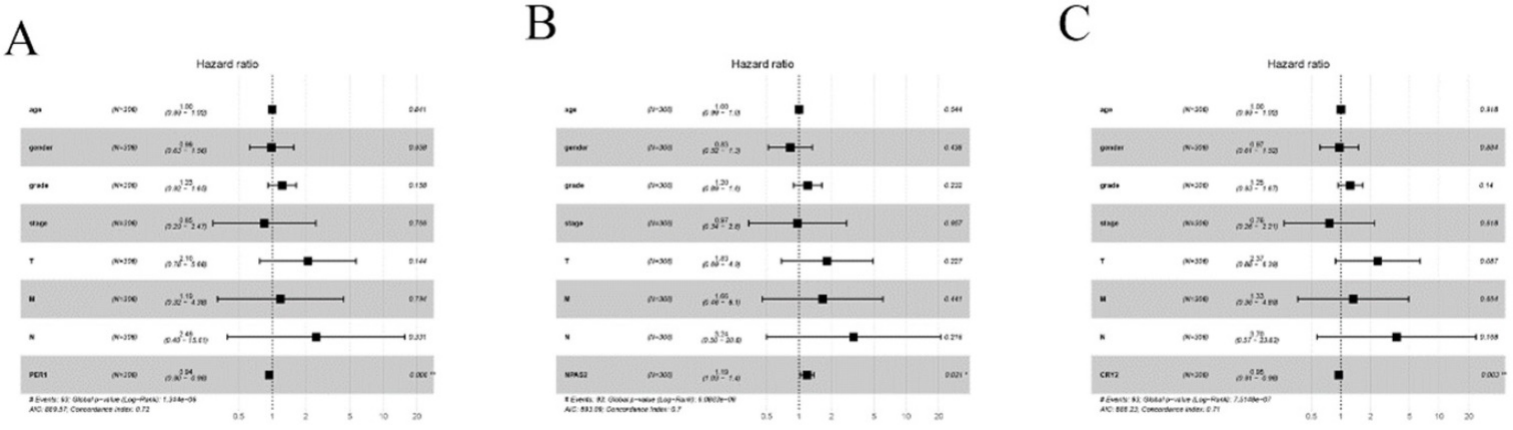
Forest plots of multiple Cox regression analysis of PER1, NPAS2, and CRY2 with significant prognostic significance. (A) PER1; (B) NPAS2; (C) CRY2. *P < 0.05; **P < 0.01.

Univariate and multivariate Cox proportional hazards regression analyses were employed to assess whether these three genes were independently related to HCC patients' prognosis. The results of univariate Cox regression analysis indicated that PER-1, CRY2, NPAS2, and the clinical stage were related with poor prognosis. The hazard ratio (HR), 95% confidence (95% CI), and P-values are reported in Table 1. Based on multivariable Cox analysis, the expression of PER-1 (HR=0.94, 95% CI=0.90-0.98, P=0.06), CRY2 (HR=0.095, 95% CI=0.91-0.98, P=0.003), and NPAS2 (HR=1.19, 95% CI=1.03-1.38, P=0.021) were independent prognosis biomarkers of HCC survival, which can be seen in the Forest plots in Figure 5. These results indicated that PER-1, CRY2, and NPAS2 were independently associated with the prognosis of HCC patients and could be used as valuable biomarkers to predict survival of HCC patients.

**Figure 6:**
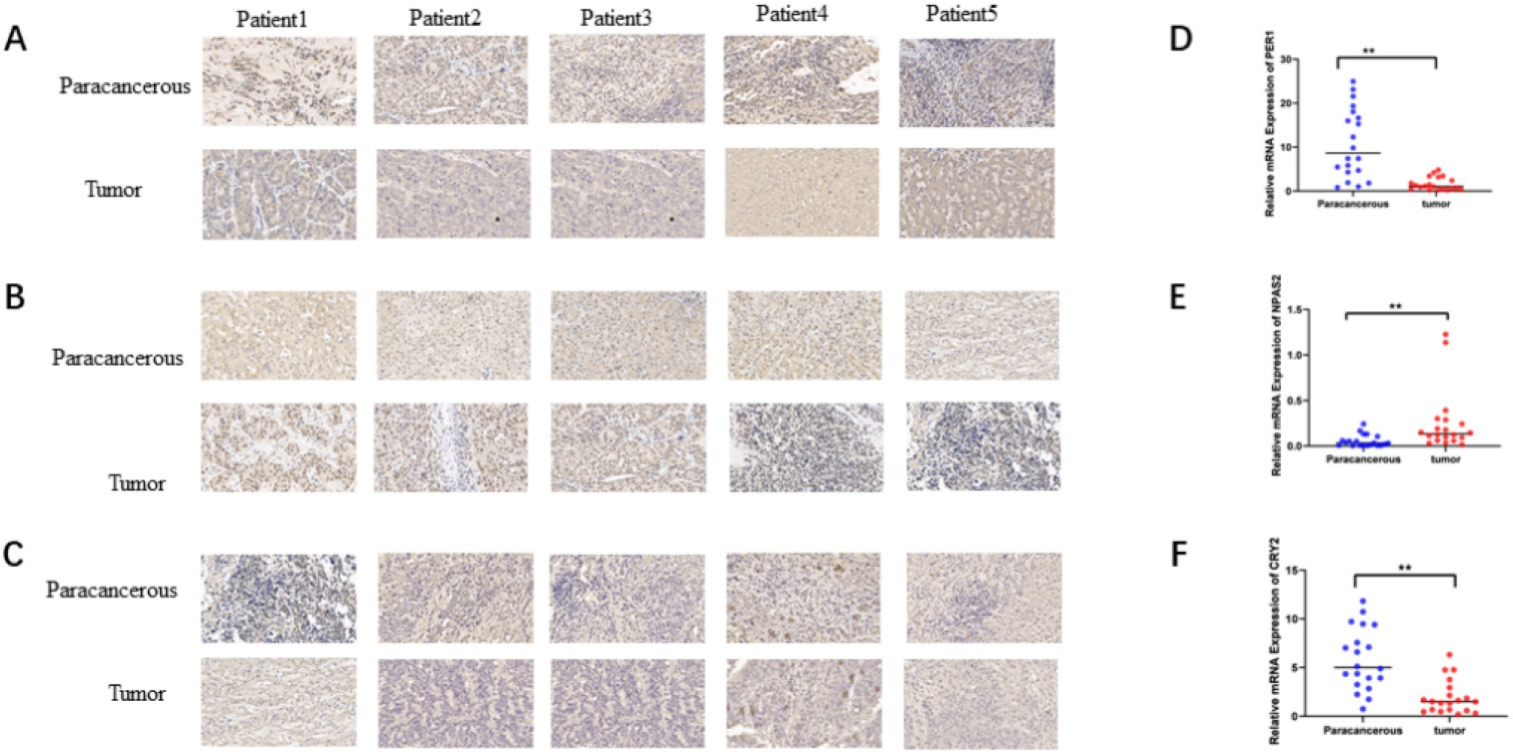
Relative expression of mRNA and protein of valuable biomarkers of circadian clock genes. A. Relative protein expression of PER1in HCC and adjacent (×40). B. Relative protein expression of NPAS2 in HCC and adjacent (×40). C. Relative protein expression of CRY2 in HCC and adjacent (×40). D. Relative mRNA expression of PER1 in HCC and adjacent. E. Relative mRNA expression of NPAS2 in HCC and adjacent. F. Relative mRNA expression of CRY2 in HCC and adjacent.

To verify the mRNA and protein levels of the three valuable genes, we examined the expression of PER-1, NPAS2, and CRY2 in twenty cases of HCC by immunohistochemistry and RT-qPCR. Detailed clinic parameters were exhibited in Table 2 (Cohort). As shown in Figure 6, we found that the expression of PER-1 and CRY2 HCC tissues was significantly higher than that in adjacent tissues, while NPAS2 was lower than that in adjacent tissues.

### Circadian clock gene expression correlated with immune infiltration in HCC

**Figure 7:**
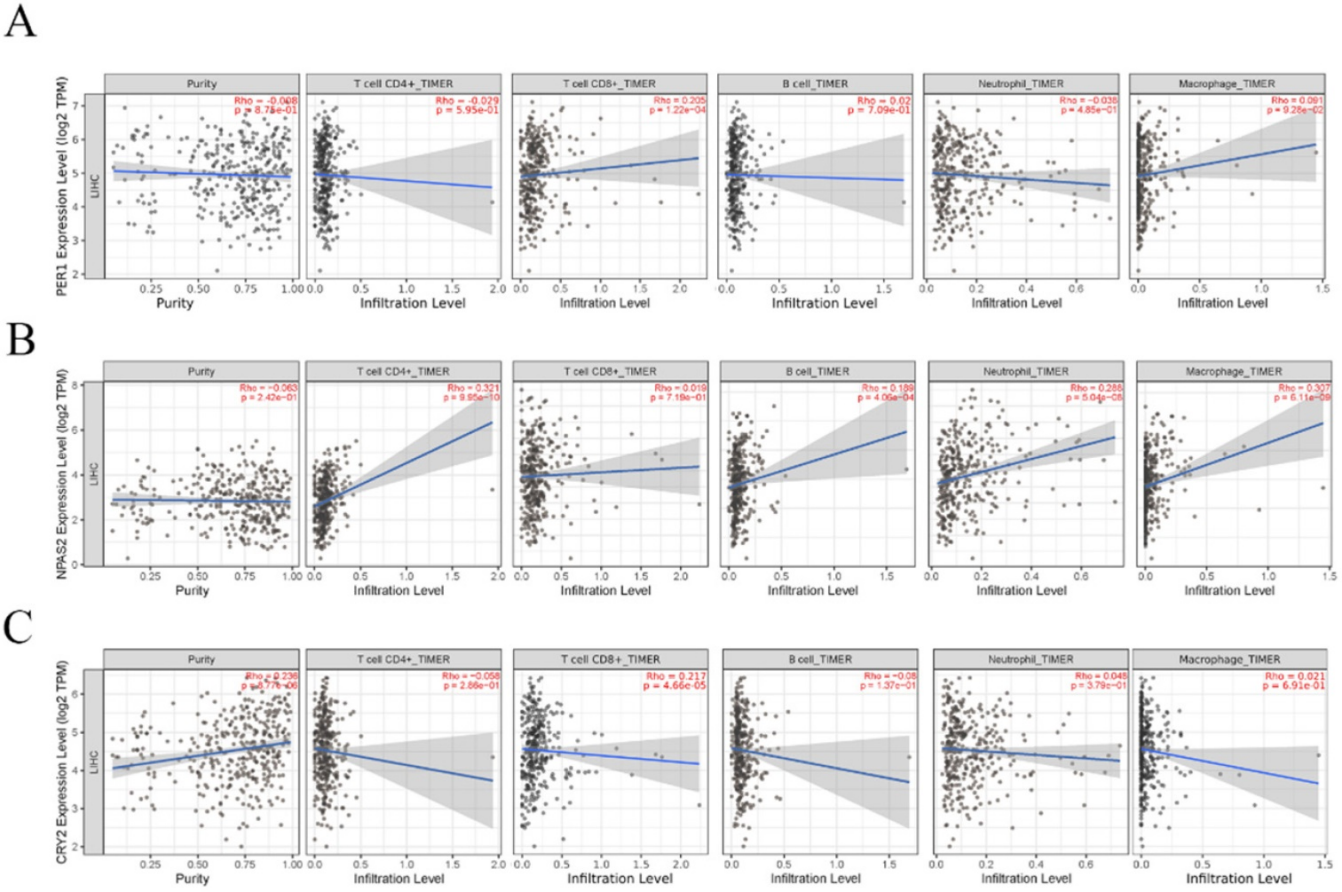
Correlation analysis between significant prognostic circadian clock members in HCC and the immune infiltration cells (CD4+ T cells, CD8+ T cells, B cells, neutrophils, and macrophages). A. Correlation analysis between PER1 expression level and the immune infiltration cells. B. Correlation analysis between NPAS2 expression level and the immune infiltration cells. C. B. Correlation analysis between NPAS2 expression level and the immune infiltration cells.

Accumulating data have shown that immune cells for tumor infiltration in the tumor microenvironment play an important role in tumor development 34, 35. However, it remains unclear how CCGs influence immune cell infiltration. Therefore, the TIMER platform was used to explore the correlation between independent prognostic biomarkers of CCGs and five type of tumor infiltration immune cells, including CD4+ T cells, CD8+T cells, B cells, neutrophils, and macrophages. The results indicated that the expression of CRY2 was positively correlated with tumor purity, whereas the expression of PER-1 and NPAS2 were not associated with tumor purity. In addition, the expression of PER-1 and CRY2 was positively correlated with immune infiltration of CD8+ T cells and the expression of NPAS2 was positively correlated with the infiltration of CD4+T cells, B cells, neutrophils, and macrophages, as shown in Figure 7.

We then identified which immune cells subtypes present in the tumor infiltration were associated with the expression of the three CCGs, PER-1, CRY2, and NPAS2 and the prognosis of HCC. By estimating the co-expression relationship of the prognostic and specific biomarkers of different tumor infiltration immune cells via the GEPIA database in Table 2, we determined that PER-1 expression was significantly correlated with NOS2 and PTGS2 (M1 macrophage), VSIG4 (M2 macrophage), and KIR3DL2 (natural killer cells); CRY2 expression was closely correlated with CD3D (T cell), IRF5 (M1 macrophages); NPAS2 expression was closely correlated with CD3D (T cells), CD86 (monocytes), IRF5 and NOS2 (M1 macrophages), and ITGAM (neutrophils). In addition, the correlation between these three CCGs and different T cell subgroups of markers were investigated, including Th1, Th1-like, Th2, Tregs, resting Tregs, effector Tregs, effector T cells, naïve T cells, effector T cells, memory T cells, resistant memory T cells, and exhausted T cells. The results showed that the expression of PER-1 was significantly correlated with Th1-like, Th17, Treg, and naïve T cells, resistant memory T cells, and general memory T cells; CRY2 expression was closely correlated with Th1, Th1-like, Th17, and Treg cells; NPAS2 was closely correlated with Th1, Th1-like, Th17, Treg, effector T cells, effector memory T cells, resistant memory T cells, exhausted T cells, resting Treg T cells, and effector Treg T cell. The above results indicated that circadian genes, PER-1, CRY2, and NPAS2 might regulate the infiltration of tumor immune cells in HCC.

The above results showed that the expression of PER-1, RORA, and NPAS2 was significantly related to immune cell infiltration in HCC and may play a crucial role in the tumor immune microenvironment of HCC.

## Discussion

The circadian rhythm, also called the circadian clock, is dominated by a series of circadian clock genes. Abnormal expression of CCGs contributes to physiological disturbances of homeostasis, which is often associated with a serious of illnesses, including asthma, solid malignance tumors 36, 37. Multiple lines of studies have revealed that the CCGs govern the multiple widespread aspects of immune functions in cancer, including immune infiltration 14. Several studies have indicated that circulating lymphocytes in the blood exhibited high-amplitude circadian rhythms in both cell counts and function measures 38, 39. A recent study also showed that the expression of the cytolytic factors granzyme B, perforin, and the cytokine IFN-γ follow a physiological circadian rhythm that is associated with the circadian rhythm of the cytolytic activity of NK cells in the spleen. CCGs control many aspects of immune functions in solid tumors. For example, Kinker et al. 40 revealed that lung cancer is closely related to the proportions of numerous subsets of lymphocytes, including CD4+ T cells, BK cells. Another independent group reported that BMAL1 was a potential biomarker for T cell-based immunotherapies in melanoma 41. However, it is still unclear about the relationship between circadian clock and immune infiltration in HCC. Hence, we carried out multi-omics tools to explore correlation between circadian clock and immune infiltration in HCC.

First, we evaluated the rhythm of 14 CCGs in liver tissues. We found that the expression of most CCGs in liver tissue is rhythmic and more than half of CCGs were dysregulated in HCC. Furthermore, we evaluated the prognosis role of the core circadian clock gene expression in HCC using Kaplan-Meier survival analysis and Cox proportional hazards regression. The result indicated that decreased expression of PER-1, CRY2, and RORC, as well as overexpression of NPAS2, contributed to poor survival of HCC patients as independent prognostic biomarkers. Previous study has shown that PER-1 is closely related to the stage and prognosis of ovarian cancer 42. In addition, genetic variants in the NPAS2 gene are associated with large tumor size, lymph node metastasis, and a poorer prognosis 43. Tumor tissues in patients with pancreatic ductal adenocarcinoma expressed a lower level of PER-1 and CRY2, which also predicts the poor OS of patients with pancreatic ductal adenocarcinoma 44. Thus, this study is consistent with previous research on other malignant tumors.

The development of immunotherapy strategies to eliminate cancer cells has led to imperative breakthroughs in a serious of solid tmors. However, the effect of immunotherapy in HCC patients is not satisfactory 45. This is mainly due to the absence of predictive markers, the lack of immune cell infiltration, and the complex TME. Recent studies have shown that immune infiltration in the TME plays a significant role in the development of HCC and affects the prognosis of patients with HCC 46. Thus, a comprehensive understanding of immune infiltration in TME contributes to a greater understanding of the potential molecular mechanisms and new strategies to improve the efficiency of immunotherapy 47. Therefore, we investigated the relationship between the CCGs and immune infiltration in HCC. A key result of the present study was that the expression of PER-1 and CRY2 was closely related to the levels of CD8+ T cells, while the expression of NPAS2 was correlated with CD4 + T cells, B cells, neutrophils, and macrophages. Collectively, our observation revealed that the expression of circadian clock genes, PER-1, CRY2, and NPAS2, was closely related to immune infiltration in HCC. In other words, we propose that chronoimmunotherapy in cancer may be exploited to improve effectiveness and reduce side effects in the clinical setting.

Our study presents the following limitations that should be taken into consideration. First, the expression of core CCGs was determined and analyzed using a database with limited clinical information, which thus, only reflects certain features rather than global alterations in expression. Second, the time of day at which samples were collected from different patients was not controlled. In fact, apart from the time of day, other factors, including environment and genetic factors, may also modulate core circadian clock genes. Therefore, the conclusions of the study may have been influenced by certain bias. In the future, further laboratory studies and clinical research need to be conducted to verify the functional features and molecular mechanisms of these genes.

## Conclusions

Our study indicated a disruption of CCGs in HCC and identified that the expression of PER-1, CRY2. And NPAS2 could be used as independent predictor of survival. These genes might be applied as candidate molecular targets for the diagnosis and therapy of HCC. Thus, chronoimmunotherapy in cancer may be exploited to improve treatment efficacy and reduce side effects in the clinical setting.

## Figures and Tables

**Table 1:** Univariate analysis of Cox proportional hazards regression analyses of PER-1, NPAS2, CRY2 and clinical features in HCC

Parameter	Univariate analysis		
	Hazard radio	95% CI	*P* value
Age	1.001697351	0.99-1.02	0.83
Gender	1.124051955	0.73-1.72	0.59
Grade	1.175434513	0.89-1.55	0.257
Stage	1.867896364	1.49-2.34	6.39E-08
T	1.827326534	1.47-2.26	4.20E-08
N	2.370582866	0.58-9.68	0.229
M	4.765986071	1.50-15.15	0.008
PER-1	0.929335985	0.89-0.97	0.001
NPAS2	1.229189017	1.08-1.40	0.001
CRY2	0.942564678	0.91-0.98	0.001

**Table 2:** Clinical and pathological characters of HCC patients in TCGA and cohort

Characteristic	TCGA (n=350)	Cohort (n=20)
**Age (%)**		
≤60 (%)	174(49.71%)	14 (70%)
>60 (%)	176(50.29%)	6 (30%)
**Gender (%)**		
Male (%)	249 (68.29%)	12(60%)
Female (%)	101 (31.71%)	8 (40%)
**ACCJ stage (%)**		
I-II (%)	259 (74%)	15 (75%)
III-IV (%)	91 (26%)	5 (25%)

**Table 3:** Correlation analysis between significant prognostic circadian clock expression and immune cell markers in HCC

Description	Gene marker	PER-1		CRY2		NPAS2	
		Cor	P	Cor	P	Cor	P
CD8+ T cell	CD8A	0.097	0.047	-0.08	0.1	0.08	0.1
	CD8B	0.016	0.75	-0.16	0.0013	0.06	0.22
T cell (general)	CD3D	0.093	0.057	-0.25	3.5E-07	0.23	3E-06
	CD3E	0.07	0.15	-0.12	0.012	0.13	0.0068
	CD2	0.02	0.69	-0.14	0.0052	0.18	0.00018
B cell	CD19	-0.03	0.54	-0.14	0.0048	0.15	0.0028
	CD79A	0.041	0.41	-0.16	0.001	0.019	0.7
Monocyte	CD86	0.094	0.056	-0.11	0.029	0.2	3E-05
	CSF1R	0.15	0.002	-0.066	0.18	0.15	0.0019
TAM	CCL2	0.076	0.12	-0.12	0.012	0.07	0.15
	CD68	0.05	0.31	-0.15	0.0025	0.26	1.1E-07
	IL10	0.053	0.28	-0.12	0.015	0.046	0.35
M1	IRF5	0.053	0.28	0.25	3.4E-07	0.41	1.3E-8
	NOS2	0.28	3.4E-09	0.42	4E-19	0.2	2.6E-5
	PTGS2	0.22	4.6E-06	-0.02	0.69	0.078	0.11
M2	CD163	0.12	0.011	-0.13	0.007	0.097	0.046
	VSIG4	0.17	0.0006	-0.08	0.081	0.039	0.42
	MS4A4A	0.2	2.6E-05	0.025	0.61	0.084	0.086
Neutrophil	CEACAM8	0.039	0.43	-0.06	0.19	0.006	0.9
	ITGAM	0.085	0.083	0.032	0.51	0.35	3.5E-13
	CCR7	0.15	0.0015	0.003	0.95	0.11	0.02
Natural Killer Cell	KIR2DL1	0.17	0.0005	0.078	0.11	0.048	0.33
	KIR2DL3	0.15	0.0024	0.041	0.41	0.058	0.23
	KIR2DL4	0.14	0.0036	0.023	0.64	0.037	0.46
	KIR3DL1	0.21	9.2E-06	0.11	0.029	0.17	0.00039
	KIR3DL2	0.1	0.033	0.046	0.35	0.078	0.11
	KIR3DL3	0.041	0.4	0.009	0.85	-0.01	0.84
